# Evaluation of drug interactions of saxagliptin with sildenafil in healthy volunteers

**DOI:** 10.1007/s00228-022-03397-w

**Published:** 2022-10-10

**Authors:** Rania Y. Mansour, Radwa ElBorolossy, Sara M. Shaheen, Nagwa A. Sabri

**Affiliations:** 1 MScs. Of Clinical Pharmacy, Faculty of Pharmacy, Ain Shams University, Cairo, Egypt; 2 Clinical Pharmacy Department, Faculty of Pharmacy, Ain Shams University, Cairo, Egypt

**Keywords:** Saxagliptin, Sildenafil, Diabetes, Sexual dysfunction, Pharmacokinetics, Pharmacodynamics, Drug interactions

## Abstract

**Purpose:**

The purpose of this study is to investigate the effect of sildenafil a CYP3A4 substrate and inhibitor on the pharmacokinetics and safety of saxagliptin.

**Methods:**

Eighteen healthy volunteers were recruited in sequential; single-center study to determine pharmacokinetic parameters of saxagliptin and sildenafil, and (AUC_0-∞_), (AUC_0-t_); C_max_; t_max_; t_½_, k_e_; k_a_ were measured using validated LC–MS/MS method. Therapeutic doses were given as follows: Sildenafil 50 mg single dose on day one, then washout period from day two till day eight. Saxagliptin 5 mg once/day was given from day 9 till day 12; then on day 13, the two drugs were co-administered. Blood samples for pharmacokinetic analysis were collected on days 1 and 13 for sildenafil and on days 12 and 13 for saxagliptin.

**Results:**

Saxagliptin ratios of T/R and 90% CI were 132.1% (122.7–142.3) for AUC_0–*t*_, and 167.6% (154.6–181.8) for C_max_. On the other hand, sildenafil pharmacokinetics were not affected. G_max_ changed from 93.7 mg/dl to 95.6 mg/dl (*P* > 0.001) and AUC_g0-t_ from 512.8 ng.h/ml to 532.75 ng.h/ml (*P* > 0.001) after co-administration of both drugs.

**Conclusion:**

Sildenafil significantly affected the pharmacokinetic parameters of saxagliptin when co-administered.

**Registration:**

This trial was registered at clinicaltrials.gov under identifier number: [NCT04170790] in November 2019.

## Introduction

Drug-drug interactions (DDIs) are one of the most common causes of medication error in developed countries, particularly in the elderly due to poly-therapy, with a prevalence of 20–40% [1]. Poly-therapy increases the complexity of therapeutic management and thereby the risk of clinically important DDIs, which can both induce the development of adverse drug reactions or reduce the clinical efficacy of the drugs. DIs cause altered pharmacological response leading to toxicity or therapeutic failure [1]. These processes are considered preventable and need intervention by improvement in diagnosing and prescribing skill [2].

There is a potential possibility of harmful effects owing to DIs; however, they may produce some beneficial effects or no effect at all. Several investigations have shown that 10–20% of the DIs have fatal consequences and are responsible for the patients’ hospitalization [3].

Identifying drug-drug interaction (DDI) is an important topic for the development of safe pharmaceutical drugs and for the optimization of multidrug regimens for significant diseases and lifelong health problem such as diabetes mellitus (DM) which is a complex chronic illness associated with hyperglycemia, occurring from deficiencies in insulin secretion, action, or both [4].

Gliptins represent a novel class of agents that improve beta cell health and suppress glucagon, resulting in improved post-prandial and fasting hyperglycemia. They function by augmenting the incretin system (GLP-1 and GIP) preventing their metabolism by dipeptidyl peptidase-4 (DPP-4). Not only are they efficacious but also safe (weight neutral) and do not cause significant hypoglycemia, making it a unique class of drugs [5]. Available gliptins are sitagliptin, vildagliptin, saxagliptin, linagliptin, and alogliptin [5].

The pharmacokinetic profile of saxagliptin has been determined in healthy subjects (saxagliptin 100–400 mg) and patients with type 2 diabetes (saxagliptin 2.5–50 mg). Systemic exposure to saxagliptin is dose-proportional, and pharmacokinetic parameters are similar for both populations studied. The time to reach maximal plasma concentration is < 2 h and the mean half-life is calculated as 2.2–3.8 h [6].

Saxagliptin is generally well-tolerated. In a phase I study, patients with type 2 diabetes received saxagliptin at doses of up to 50 mg (i.e., 10 times the recommended therapeutic dose) for 2 weeks. No apparently dose-related adverse events or laboratory abnormalities (including effects on the corrected QT interval) were observed. The most frequently reported adverse events in phase III clinical trials evaluating saxagliptin were headache, upper respiratory tract infection, urinary tract infection, nasopharyngitis, and back pain [7]. Saxagliptin is primarily metabolized by CYP3A4/3A5; data from a study administering radio-labeled saxagliptin indicated that 5-hydroxy saxagliptin is the major metabolite of saxagliptin in humans [7].

Type 2 diabetes mellitus is associated with endothelial dysfunction and a risk for systemic atherosclerosis and cardiovascular events. Hyperglycemia in diabetes induces oxidative stress, which is a trigger of endothelial dysfunction by reducing nitric oxide (NO) bioavailability [8].

Patients with diabetes are three times more likely to develop erectile dysfunction (ED), and a longer duration of diabetes is strongly associated with ED [9]. The pathophysiology of diabetic impotency is multifactorial, and no single etiology is at the forefront. Following are the proposed mechanisms of ED in diabetic patients: advanced glycation end-products (AGEs) and increased levels of oxygen free radicals, impaired NO synthesis, increased endothelin and endothelin-B receptor binding sites, up-regulated RhoA/Rho-kinase pathway, and neuropathic damage impaired cGMP-dependent protein kinase-1 (PKG-1) [10].

Oral PDE-5 inhibitors are the first-line treatments for ED. They help to maintain the erection by enhancing the vasodilatory effects of endogenous nitric oxide. Sildenafil citrate has been the drug of choice for the treatment of ED of organic, psychogenic, or mixed aetiologies [11].

Evidence is provided for CYP3A4 and to a lesser extent CYP2C9-mediated metabolism of sildenafil. There is the possibility that elevated plasma concentrations of sildenafil could occur with co-administration of other drugs metabolized by CYP3A4 [11].

Sildenafil was the first selective inhibitor of cGMP-specific PDE5 available on the market as oral therapy for ED [12]. Sildenafil is rapidly absorbed, with maximum observed plasma concentrations (C_max_) reached within 30–120 min (median time 60 min) after oral administration under fasting conditions. The mean absolute oral bioavailability is 41% (range 25–63%). The area under the concentration–time curve (AUC) and Cmax increase proportionally with the dose over the recommended oral dose range (25–100 mg) indicating a dose-proportional rate and extent of absorption. When sildenafil is taken after a heavy and fatty meal, the rate of absorption is reduced with a delay in T_max_ and a mean reduction in C_max_ by 29% [12].

The mean steady-state sildenafil volume of distribution is 105 L, indicating high distribution into the tissues. The total sildenafil body clearance is 41 L/h with a resultant terminal half-life (t1/2) of 3–5 h. Sildenafil is cleared predominantly by the CYP3A4 (major route) and CYP2C9 (minor route) hepatic microsomal isoenzymes. The major circulating metabolite, resulting from N-demethylation of sildenafil, has a PDE selectivity profile similar to sildenafil and an in vitro potency for PDE5 approximately 50% of the parent drug. Plasma concentrations of this metabolite are approximately 40% of those found for sildenafil. The N-desmethyl metabolite is further metabolized, with a t1/2 of approximately 4 h [13]. The drug and its major circulating N-desmethyl metabolite are bound to plasma proteins in the amount of 96%, and binding is independent of total drug concentrations [14].

In addition to that the two study drugs are metabolized by CYP3A4/3A5, both affect the smooth muscles and nitric oxide where Glucagon-like peptide 1 receptor (GLP-1R) is widely expressed in cardiovascular systems such as endothelium, vascular smooth muscle, and cardiac atrium. GLP-1R activation on endothelial cells has been shown to be able to increase cyclic adenosine monophosphate (cAMP), followed by the activation of Protein kinase A (PKA) and endothelial nitric oxide synthase (eNOS) [15].

The activation of eNOS subsequently results in the release of nitric oxide (NO) and vessel relaxation. Studies in humans also confirmed the vasodilatory effect of GLP-1. GLP-1 analogs are also able to reduce blood pressure by increasing urinary sodium excretion (www.fda.gov), while sildenafil is a highly selective inhibitor of PDE type 5, and it enhances NO-mediated relaxation of human corpus cavernosum in vitro. Sildenafil, by inhibiting phosphodiesterase, increases the intracellular concentrations of cyclic guanosine 3′, 5′ monophosphate (cGMP), causing an amplification of the endogenous NO-cGMP signaling pathway which may result in an exaggerated pharmacodynamic effect in the form of hypotension [16].

Regarding analytical assays, literature data showed a highly sensitive, selective, and specific LC/MS/MS approach for estimating a combination of saxagliptin (SAX) and dapagliflozin (DAP) in rat plasma. The chromatographic separation was performed on a C_18_ column (150 mm, 4.6 mm, 3.6 m) with gradient elution using 0.01 percent ammonia solution and acetonitrile as the mobile phase. The ion transitions were measured in both positive and negative polarity. A solid phase extraction procedure was applied for sample clean-up. The technique demonstrated high linearity in the range of 0.2 to 80 ng/mL for SAX and 5 to 2000 ng/mL for DAP [17].

Consequently, the objective of this study was to undergo a pharmacokinetic study to investigate the incidence of potential pharmacokinetic interaction between steady-state saxagliptin and sildenafil through investigation of changes, if present, of the pharmacokinetics and pharmacodynamics parameters of both drugs administered alone and in combination.

## Materials and methods

### Materials

#### Chemicals and reagents

Purified water for LC/MS/MS grade, human plasma (Vacsera blood bank), methanol (SIGMA Aldrich, Germany), Acetonitrile (Scharlab, Spain), Dichloromethane (Fisher Scientific, UK), diethyl ether (Scharlab, Spain), formic acid (Scharlab, Spain), ammonium formate (SIGMA Aldrich, Germany), and ammonia solution (Fisher Scientific, UK).

#### Equipment’s

Adjustable pipettes (P200 and P1000), disposable plastic pipettes tips—Labtip Yellow (range 5–200 µL) and Labtip Blue (range 200 1000 µL), disposable glass test tubes 120 × 12 mm, vortex mixer (Boeco, Germany), vacuum pump (Boeco, Germany), PH-meters (Boeco, Germany), water purifier (Purelab option-R7ELGA, U. K.), Sonicator (Crest, USA), analytical balance (Sartorius, USA), LC–MS/MS Agilent 6410B Triple Quad, USA.

### Methods

#### Chromatographic conditions

An LC–MS/MS method was developed in-house for quantification of saxagliptin and sildenafil in plasma over a calibration range of 0.1–60 ng/ml for saxagliptin and 1–500 ng/ml for sildenafil. Mobile phase composition is 25 mM ammonium acetate: methanol 25:75 V/V. The flow rate was set at 0.7 ml/min. Injection volume was set at 2.5 ul. MS/MS 6410B detector was operated at ESI positive mode, m/z was 316.2 → 180.2, 304.2 → 154 for saxagliptin and vildagliptin (internal standard), 475 → 100, and 489 → 151 for sildenafil and vardenafil (internal Standard), respectively.

Fragmentor energy was set at 100 for both saxagliptin and vildagliptin (internal standard) and 135 for both sildenafil and vardenafil (internal standard). The collision energy was set at 20 and 24 for saxagliptin and vildagliptin (internal standard) and 25 and 55 for sildenafil and vardenafil (internal standard) respectively.

#### Sample preparation

A solid-phase extraction method was used for extraction of the analyte from 250 ul human plasma sample by using an Oasis MCX cartridge after the addition of 25 ul of vildagliptin 1 ug/ml and vardenafil 1 ug/ml (internal standards). Before transferring the plasma sample to the MCX cartridge, acidification of the plasma sample with 25 0ul of (formic acid 2%) was performed. The sample was washed with 200 ul of 2% formic acid in water followed by 200 ul of methanol. After that, elution with 150 ul of 5% ammonia in acetonitrile (75 ul × 2) was done. Then 350 ul of water was added to the eluent and vortexed. After that, the diluted eluent was transferred to a vial insert, and 2.5 μl was injected into LC/MS/ MS system for analysis.

#### Bioanalytical method validation

Concentrations of saxagliptin and sildenafil in plasma were determined by analyzing the sample on a validated LC/MS/MS bioanalytical method. The limit of quantification was defined considering the method sensitivity, the precision, and the accuracy. To evaluate precision and accuracy, specific quality control samples were included in the validation procedure. Although sensitivity was good enough to quantify even lower values, measures were taken to guarantee an LLOQ near to 1–3% of the anticipated C_max_. The LLOQ validated under the condition found during the pre-study validation was of 0.1 ng/ml for saxagliptin and 1 ng/ml for sildenafil.

Peak area ratios of varying amounts of saxagliptin and sildenafil in plasma in the required concentration range should be highly linear (R^2^ not less than 0.998). The results of intraday precision C.V.% should be in accordance with FDA guidelines. Accuracy and precision were assessed at three different concentrations in the range of predicted drug concentrations on within and between-day basis. The lower limit of quantitation must show adequate quantitation limit to cover small drug concentration ranges during the elimination phase. Quality control samples concentrations were defined as presented in (Table 1):

**Table 1:** Quality control samples definition for saxagliptin and sildenafil

**QC type**	**QC code**	**Range definition**	**Saxagliptin defined value** **Conc. (ng/ml)**	**Sildenafil defined value** **Conc. (ng/ml)**
Low QC sample	QCA	3 × LLOQ	0.3	3
Medium QC sample	QCB	Average between low and high QC	25	200
High QC sample	QCC	75–90% of the highest calibration	50	400

#### Quantitation

Unknown drug concentrations in plasma samples withdrawn calculated using the following equation: *y* = ax + b, where; Y: response ratio, X: unknown concentration of drug in plasma samples, a: calibration curve slope, and b: Y-Intercept.

#### Study ethics

This study was conducted in accordance with the ICH and GCP guidelines adopted by the European agency for the evaluation of medicinal products (EMEA), and after Ethics Committee approval on the study protocol by Faculty of Pharmacy, Ain Shams University (Study Code:57) and additionally approval of Ethics Committee of Drug Research Center (Study Code: SAX-RES-BS-0418–0010). Essential documents and records were all archived according to drug research center (DRC) internal procedures for authorized direct access.

Written informed consents were signed by the participant and clinical investigator, and all study aspects were discussed with participants before starting of screening. There were no obligations on volunteers to continue the study if they did not want to.

Clinical investigator, study director (principal investigator), licensed physicians responsible for physical examination and following up of the subjects for the appearance of any side or adverse effects, measurement of vital signs throughout the study including blood pressure, pulse rate, body temperature, respiratory rate before and all over the study, and registered nurses were responsible for blood sampling.

#### Study design

The study was a single-center, sequential, single-blind, and interventional study that was conducted on 18 healthy volunteers to determine potential pharmacokinetic interaction between saxagliptin and sildenafil. Therapeutic doses were given to healthy volunteers as follows: on the 1st day all volunteers were administered a single dose of sildenafil 50 mg, and a washout period was carried out from the 2nd to the 8th day of the study, followed by administration of saxagliptin 5 mg once/day from the 9th to 12th day, then on the 13th day both drugs.

#### Inclusion criteria

Eighteen healthy volunteers aged 18–45 years, within the normal range of body mass index, normal physical health, physical examination, vital signs, clinical laboratory test, and no history of contribution in any pharmacokinetics study. Subjects should be non-smokers and should not have any history of drug or alcohol abuse.

#### Exclusion criteria

Included history or presence of significant physical or psychological diseases, history of sensitivity or allergy to any of the drugs in the study, gastrointestinal tract problems, auto-immune diseases, kidney diseases or kidney dysfunction, central nervous system diseases, diabetics, hepatic disease, hematological abnormalities, respiratory diseases, alcohol intake or drug abuse history, and positive HIV-I. Administration of over-the-counter drugs and herbal remedies was not allowed for 2 weeks before screening or participation in other clinical trials or donated blood in the past 3 months.

#### Blood sampling and drug analysis

Serial blood samples [5 ml each] for pharmacokinetic analysis were collected on days 1 and 13 for sildenafil analysis as well as on days 12 and 13 for saxagliptin analysis at the following times: pre-dose and 0.25, 0.5, 1, 1.5, 2, 2.5, 3, 4, 6, 8, 10-, 12-, 18-, and 24-h post-dose. Blood samples were collected into tubes containing EDTA disodium as an anticoagulant slightly shaken and centrifuged at approximately 4000 r.p.m. for 10 min. After centrifugation, plasma samples were transferred directly into a 5 ml-plastic tube. These samples were immediately stored at the study site in a freezer at a nominal temperature − 80 °C until analysis. The label of the collecting tubes had the study’s code number, subject number, study period, and the designated sample number. The total amount of blood loss during the whole study did not exceed 225 ml.

#### Pharmacokinetic parameters

Pharmacokinetic parameters were determined and calculated. The main study outcome measures were C_max_, T_max_, AUC_0–t_, AUC_0–∞_, t_½_, k_e_; k_a_, and CL/F.

#### Pharmacodynamic parameters

Blood glucose levels were measured for all volunteers on the ninth and the thirteenth day before dosing and at 0.5, 0.75, 1, 1.5, 2, 2.5, 3, 12, and 24 h; the main outcome measures were maximum plasma glucose concentration (G_max_) and the area under the blood glucose concentration–time curve (AUCg_0-t_). Blood pressure (systolic and diastolic) and heart rate were measured before dosing and at 2, 4, 6, 8, and 10 h after drug administration.

#### Safety and tolerability

Blood pressure, pulse rate, and body temperature together with blood glucose levels were reported all over the study. Moreover, possible side and/or adverse reactions or events to the study formulations were recorded to assure the safety and tolerability of different drug administrations.

#### Clinical case reports

Subject medical histories, physical examination, and clinical laboratory tests were reported. The study including blood pressure, pulse rate, body temperature, respiratory rate before and all over the study, and registered nurses were responsible for blood sampling.

#### Statistical analysis

The pharmacokinetic analysis was performed using the SAS program. All results were expressed as mean ± SD. Paired T-Test was used to compare results, and the level of significance was considered at (*P* < 0.05). GLM procedure was used to calculate ratios of T/R and 90% CI for C_max_, AUC_0–t_, AUC_0–∞_, and t_½_. Bioequivalence acceptance criteria are based on the 90% confidence interval for the ratio of difference of the test and reference products C_max_ and AUC falling within 80 to 125%.

Calculations were done based on Schuirman’s two one-sided T-Tests procedure using the ± 20 rule for assessment of bioequivalence. The sample size should be large enough to provide a power (*ϕ* = 1-β) of 80% for the detection of a difference of the magnitude at least 20% of the unknown reference mean. Significance level α (type I error) equal to 0.05 and β (type II error) equal to 0.2.

The sample size to provide a power of 80% for detection of a difference of the magnitude at least 20% of the unknown reference mean should be equal to/or greater than 6 subjects.

## Results

The collected demographic data of the volunteers including age, gender, height, weight, and BMI of the eighteen healthy males were as follows; an average age of 29.38 years, an average height of 173.7 cm, an average body weight of 27.5 kg, and an average body mass index of 25.34 kg.m2.

**Fig. 1:**
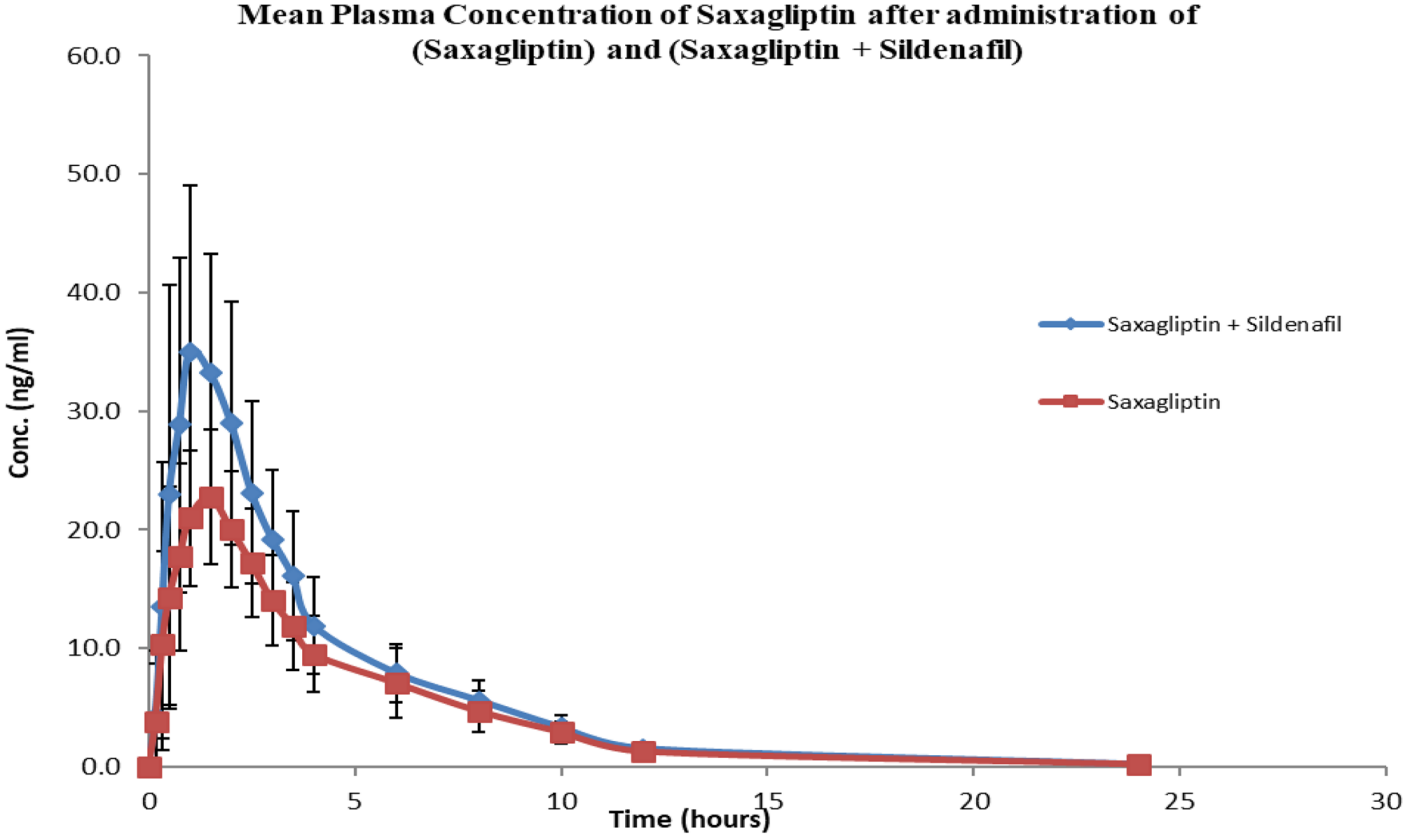
Plasma concentration levels of Saxagliptin (ng/ml) alone and after concomitant administration of multiple oral doses of Saxagliptin 5mg tablet and single oral dose of Sildenafil 50mg Tablet

Co-administration of saxagliptin and sildenafil resulted in an increase in the average C_max_ of saxagliptin from (26.35 ng/ml) to 44.679 ng/ml (*P* < 0.001) as shown in Fig. 1. AUC_0-∞_ (112.719 ng.h/ml) was also increased to 149.710 ng.h/ml, and AUC_0-t_ (111.817 ng.h/ml) was significantly increased to 148.811 ng.h/ml (*P* < 0.001). T_max_ insignificantly decreased from 1.24 h to 1.11 h (P > 0.001). However, saxagliptin and other pharmacokinetic parameters were not affected by sildenafil co-administration.

Regarding sildenafil pharmacokinetics parameters, the average value of C_max_ (301.969 ng/ml) decreased to 274.413 ng/ml, a slight increase in T_max_ value from 0.977 h to 1.264 h, and a decrease in AUC_0-t_ value from 977.363 ng.h/ml to 930.233 ng.h/ml after co-administration with saxagliptin; however, these changes were not statistically significant (*P* > 0.001). In addition, there was no significant change in the pharmacodynamic parameters G_max_, AUCg_0-t_, and blood pressure after the co-administration of 2 drugs (*P* > 0.001). Smoking did not show any statistically significant impact on the pharmacokinetics of both studied drugs (*P* > 0.001).

Pharmacokinetic point of estimates at 90% CI was calculated, and data is represented in Tables 2 and 3 for saxagliptin and sildenafil, respectively. Saxagliptin C_max_, AUC_0-t_, and AUC_0-inf_ showed pharmacokinetic interaction while other parameters did not show any significant change; on the other hand, sildenafil pharmacokinetic parameters did not show any significant change as represented in Tables 2 and 3.

**Table 2:** Ratios of T/R and 90% CI of saxagliptin following administration of multiple oral doses of saxagliptin 5 mg tablet and a single oral dose of sildenafil 50 mg tablet with multiple doses of saxagliptin 5 mg tablet to 18 volunteers

**Parameter**	**Point estimate**	**Lower CI**	**Upper CI**
**C**_**max**_	**167.7%**	**154.7%**	**181.8%**
**AUC**_**0-t**_	**132.1%**	**122.7%**	**142.3%**
**AUC**_**0**_-_**inf**_	**131.9%**	**122.4%**	**141.9%**
**T**_**1/2**_	**95.1%**	**91.9%**	**98.5%**
**MRT**	**91.6%**	**88.2%**	**95.3%**

*C*_*max*_ maximum plasma concentration,* T*_*1/2*_ time to reach half plasma concentration, *AUC*_*0-t*_ area under the plasma concentration–time curve, *AUC*_*0-inf*_ area under the curve, *CI* confidence intervals, *MRT* mean residence time

**Table 3:** Ratios of T/R and 90% CI of sildenafil following administration of a single oral dose of sildenafil 50 mg tablet and single oral dose of sildenafil 50 mg tablet with multiple doses of saxagliptin 5 mg tablet to 18 volunteers

**Parameter**	**Point estimate**	**Lower CI**	**Upper CI**
**C**_**max**_	**92.3%**	**78.9%**	**108.9%**
**AUC**_**0-t**_	**102.6%**	**85.9%**	**123.1%**
**AUC**_**0**_-_**inf**_	**102.8%**	**85.9%**	**123.1%**
**T**_**1/2**_	**104.5%**	**95.9%**	**113.8%**
**MRT**	**106.9%**	**96.9%**	**118.1%**

*C*_*max*_ maximum plasma concentration, *T*_*1/2*_ time to reach half plasma concentration, *AUC*_*0-t*_ area under the plasma concentration–time curve, *AUC*_*0-inf*_ area under the curve, *CI* confidence intervals, *MRT* mean residence time

**Fig. 2:**
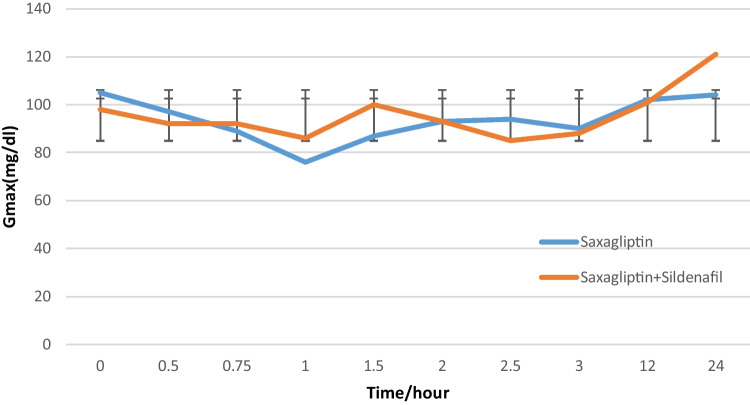
Saxagliptin G_max_ after Multiple dose administration “Saxagliptin 5mg Tablet” and “Saxagliptin 5mg Tablet + Sildenafil 50mg Tablet” (*P *> 0.001)

Regarding pharmacodynamic parameters, maximum glucose concentration (G_max_) changed from 93.7 mg/dl when saxagliptin is administered in multiple doses to 95.6 mg/dl after the administration of both drugs (*P* > 0.001), as shown in Fig. 2, also the change in AUCg_0-t_ from 512.8 ng.h/ml after saxagliptin multiple dosing compared to 532.75 ng.h/ml after co-administration of both drugs was statistically non-significant (*P* > 0.001).

Blood pressure and pulse rate were also comparable after the co-administration of the 2 studied drugs (*P* > 0.001).

### Safety and tolerability

About drug’s adverse effects, there was an increased incidence of reported headache after co-administration of the 2 drugs (*n*- = 8, 44.4%) compared to only (*n* = 6, 33.3%) in those who administered sildenafil alone and (*n* = 0, 0%) who administered saxagliptin alone.

## Discussion

The safe administration of sildenafil with saxagliptin in diabetic-induced erectile dysfunction was not studied. The possibility of pharmacokinetic interactions may occur as the two drugs are metabolized by hepatic CYP3A4, and their co-administration may affect their plasma concentrations, and dose adjustment may be required; in addition, both affect the smooth muscles and nitric oxide where Glucagon-like peptide 1 receptor (GLP-1R) is widely expressed in cardiovascular systems such as endothelium, vascular smooth muscle, and cardiac atrium. GLP-1R activation on endothelial cells has been shown to be able to increase cyclic adenosine monophosphate (cAMP), followed by the activation of Protein kinase A (PKA) and endothelial nitric oxide synthase (eNOS) [15].

The current study was mainly conducted to highlight the drug-drug interactions that might occur due to the co-administration the saxagliptin and sildenafil. Since the weak control on drug dispensing in developing countries of many of the prescribed drugs can be taken over the counter. Even when effective treatment options exist, poor understanding of the safe and effective use of these medications leads to adverse drug reactions and/or loss of efficacy.

The current study results showed that saxagliptin was rapidly absorbed after oral administration with C_max_ 26.35 ng/ml and T_max_ 1.24 h, where the obtained pharmacokinetic and pharmacodynamic parameters are comparable to those stated in the literature.

Many drug interaction studies were conducted to evaluate saxagliptin’s influence on the pharmacokinetics of co-administered drugs and vice versa [18].

An open-label, non-randomized, and sequential study with 24 healthy subjects evaluated saxagliptin interaction with simvastatin, a 3-hydroxy-3-methylglutaryl coenzyme A (HMG-CoA) inhibitor, a substrate for CYP3A4/5. In this study, the effects of once-daily simvastatin 40 mg on the steady-state pharmacokinetics of once-daily saxagliptin 10 mg and the effects of saxagliptin on simvastatin pharmacokinetics were investigated, and the results revealed that the C_max_ of saxagliptin increased by 21% after co-administration of simvastatin compared with saxagliptin administration alone, whereas, the overall systemic exposure to saxagliptin [AUCs] was not affected. On the other hand, co-administration of saxagliptin with simvastatin did not affect the geometric mean C_max_ or AUCs values for simvastatin [19].

It is worthy to mention that saxagliptin was co-administered with simvastatin, a substrate that is considered to be sensitive to changes in CYP3A4 activity; there was no impact of saxagliptin on simvastatin pharmacokinetic parameters. Because the study was conducted under steady-state conditions, the data support the conclusion that saxagliptin neither induces nor inhibits CYP3A4 activity. Simvastatin is also a substrate of P-glycoprotein [19].

The lack of a clinically meaningful change in simvastatin and simvastatin acid pharmacokinetics also indicates that saxagliptin does not modulate the activity of these transporters. While there was a small increase in saxagliptin exposure when saxagliptin was co-administered with simvastatin, there was no clear corresponding change in the exposure to 5-hydroxy saxagliptin, suggesting that alteration of CYP3A4 metabolism may not be the mechanism for the small increase in parent exposure [19].

In another study, the interaction with saxagliptin with diltiazem a moderate inhibitor of CYP3A4/5 was investigated, and the potential of diltiazem to reduce the metabolism of saxagliptin was studied. Patients with T2DM often have cardiovascular disease, making concomitant use of saxagliptin and diltiazem by 63 and 109%, respectively. So, the pharmacokinetics of saxagliptin after co-administration with diltiazem compared with administration of saxagliptin alone was assessed, and the results revealed that the geometric mean C_max_ and AUC_0-inf_ of 5-hydroxy saxagliptin decreased by 43 and 34%, respectively, after co-administration of diltiazem [20].

Sildenafil is metabolized primarily by the cytochrome P450 enzyme 3A4, which is the principal enzyme responsible for the oxidative metabolism of most drugs. The interactions between sildenafil and other drugs that are metabolized by CYP3A4 should be considered, because enzymes that compete with sildenafil for 3A4, especially those that are inhibitors of the enzyme, could cause unwanted pharmacological effects such as elevated and prolonged serum concentrations of sildenafil [21].

Clinical research on strong inhibitors of cytochrome P450, specifically, co-administration of sildenafil with potent 3A4 inhibitors such as azole antifungal agents, macrolide antibiotics, and protease inhibitors, suggests caution with dosing [21].

It was suggested that administration of sildenafil with inhibitors of 3A4 should consider using a lower starting dose [21], and yet, others suggest that those on 3A4 inhibitors should not exceed the usual minimum dosage of 25 mg in any 48 h period [21]. Sildenafil itself is a weak inhibitor of 3A4 and may occasionally interfere with the degradation of substrates cleared by that enzyme system [21].

In the current study when saxagliptin and sildenafil were co-administered, saxagliptin ratios of T/R and 90% CI were 132.1% (122.7–142.3) for AUC_0–t_, and 167.6% (154.6–181.8) for C_max_. C_max_ was increased from 26.35 ng/ml to 44.679 ng/ml with *P* < 0.001; in addition, AUC_0-∞_ and AUC_0-t_ were also increased from 112.719 ng.h/ml to 149.710 ng.h/ml and from 111.817 ng.h/ml to 148.811 ng.h/ml, respectively with *P* < 0.001. Other drug-drug interaction studies did not reveal any clinically relevant alterations in saxagliptin pharmacokinetics, nor did saxagliptin affect the pharmacokinetics of any of the co-administered drugs that were tested [6].

In obese Zucker, rat saxagliptin increased NO synthesis and reduced peroxynitrite (ONOO-) production; this effect was observed before the hypoglycemic action. Moreover, saxagliptin was able to stimulate NO release from isolated aorta rings, of about 18%, with a contemporary peroxynitrite reduction. The NO/ONOO- rate raised of about 40% [22]. Studies in humans also confirmed the vasodilatory effect of GLP-1. GLP-1 analogs are also able to reduce blood pressure by increasing urinary sodium excretion [22].

Treatment monitoring is an essential strategy for reaching therapeutic goals as a result of monitoring patients’ medication levels in order to avoid sub-therapeutic or hazardous drug levels [23].

Diabetic patients are highly susceptible to the severity and incidence of COVID-19; antidiabetic agents may interact with antiviral drugs, and other therapeutic agents used in COVID-19 management [24]; from which sildenafil may have a potential therapeutic role regarding the need for invasive mechanical ventilation in COVID-19 patients. Thus, caution is required when selecting drug treatment to avoid unfavorable potential adverse events or lack of therapeutic efficacy [24].

Regarding the potential of pharmacodynamic interaction of saxagliptin and sildenafil, the current study results showed no significant changes in the related pharmacodynamic parameters where Gmax changed from 93.7 mg/dl when saxagliptin is administered in multiple doses to 95.6 mg/dl after the administration of both drugs (*P* > 0.001), also the change in AUCg0-t from 512.8 ng.h/ml after saxagliptin multiple dosing compared to 532.75 ng.h/ml after co-administration of both drugs was statistically non-significant (*P* > 0.001).

Also, regarding the safety of the concomitant administration of the two drugs, both blood pressure and pulse rate were also comparable (*P* > 0.001) confirming the safety of the co-administration of the 2 studied drugs.

## Conclusions

In the current study, the maximum concentration of saxagliptin was significantly increased with sildenafil co-administration as well as AUC_0-t_ and AUC_0-∞_ by 69.5%, 33.08%, and 32.82%, respectively. Saxagliptin ratios of T/R and 90% CI were 132.1% (122.7–142.3) for AUC_0–*t*_ and 167.6% (154.6–181.8) for*C*_max_. Saxagliptin average C_max_ (26.35 ng/ml) was increased to 44.679 ng/ml (*P* < 0.001) and AUC_0-t_ (111.817 ng.h/ml) was increased to (148.811 ng.h/ml) (*P* < 0.001). In clinical terms, sildenafil’s effect on saxagliptin concentration and bioavailability was clinically significant as the C_max_ and AUC_0–t_ increase was not matching with acceptable bioequivalence limits 80–120%. Saxagliptin did not affect pharmacokinetic parameters of sildenafil neither statistically nor clinically. On the other hand, the incidence of reported headache increased after co-administration of the 2 drugs (*n* = 8, 44.4%) compared to only (*n* = 6, 33.3%) in those who administered sildenafil alone and (*n* = 0, 0%) who administered saxagliptin alone. However, there was no significant difference in blood pressure values measured in any of the three phases. In addition, there was no significant change in the pharmacodynamic parameters G_max_, AUCg_0-t_, and blood pressure after the co-administration of 2 drugs (*P* > 0.001).

Moreover, there was no pharmacodynamic interaction between the two drugs, which can give the space for more research in the cardioprotective and blood pressure lowering effects of saxagliptin specially in patients with pulmonary hypertension and diabetes. In conclusion, the use of sildenafil for the management of erectile dysfunction in diabetic patients receiving saxagliptin seems to be a safe approach but should be taken with caution. Also, it is recommended to investigate the effects of sildenafil on saxagliptin active metabolite 5-hydroxy saxagliptin to support the results of this study about the safety of co-administration of the two drugs.

## Data Availability

Essential documents and records were all archived according to drug research center (DRC) internal procedures for authorized direct access.
